# Editorial: Public health promotion and medical education reform, volume III

**DOI:** 10.3389/fpubh.2025.1730130

**Published:** 2025-11-21

**Authors:** Jing Tian, Junhai Zhou, Xiaoyun Zeng, Jian Chen

**Affiliations:** 1 School of Basic Medical Sciences, Guilin Medical University, Guilin, Guangxi, China; 2 Department of Foreign Languages, Guilin Medical University, Guilin, Guangxi, China; 3 Department of Public Health, Guilin Medical University, Guilin, Guangxi, China

**Keywords:** public health, medical education reform, public health education, trends and challenges, local/regional medical school

## 1 Introduction

Since the outbreak of the COVID-19 pandemic, governments and local authorities have addressed this global crisis by increasing investment, making public policies, enhancing individual health awareness, and improving public health facilities and services (1). Indisputably, the coronavirus pandemic has not only contributed to considerable progress in the field of public health and medical education, but also exposed a host of issues within the public health system. After years of public health system reform, disseminating health knowledge, optimizing basic health service delivery, cultivating medical talents, and reforming medical education still need continuous exploration.

## 2 Health promotion and health education

Health promotion refers to activities that enable individuals and communities to improve their health and overall wellbeing. A fundamental and integral component of health promotion is health education. Specifically, individuals and communities health outcomes are determined by the interplay of social, economic, and cultural factors, which can be positively shaped by effective health education (2).

Over the past decades, the significance of health education and prevention has been increasingly recognized. However, existing health education systems face multiple limitations, including inequalities between developed and developing countries as well as between urban and rural areas. For example, despite substantial improvement in the availability of prevention tools and effective treatment, responses to the sexual and reproductive health remain suboptimal, particularly in some regions of Africa (3). An institution-based cross-sectional study demonstrated that the pre-exposure prophylaxis (PrEP) remains under-utilized among female sex workers in Addis Ababa, Ethiopia. This is attributed to their limited knowledge of PrEP, lack of targeted education, and restricted access to PrEP services (Berhe et al.). Furthermore, it is striking that even medical students in Pereira, Colombia, exhibited a high prevalence of risky sexual behaviors, with only one third expressing willingness to undergo sexually transmitted infections (STI) screening (Loaiza-Guevara, et al.). Beyond sexually transmitted infections, maternal mortality and severe morbidity are also prominent women's health issues. In developing countries, the maternal mortality rates are even 15 times higher than those in developed nations (4). Derbew et al. demonstrated that husbands' limited knowledge of obstetric danger signs in Ethiopia may contribute to the delays in recognizing the danger signs and seeking timely health care.

In addition, health education interventions have been shown to play a major role in the prevention and treatment of chronic diseases including cancer. Currently, cancer screening services are offered by medical institutions at all levels. However, due to insufficient education and limited awareness of cancer, only a minority of the urban residents are willing to undergo cancer screening, with the majority opting for county-level or lower-level medical institutions (Chai et al.).

Visual impairment (VI) is another global health concern, ranking behind only cardiovascular disease, cancer, and diabetes. Although VI can be prevented or slowed with timely intervention, eye care has been neglected over the years (5). A report from the developed country U.S. puts forward suggestions that healthcare workers provide relevant education to individuals experiencing visual change, thereby effectively preventing VI within communities, neighborhoods, and broader society (Powers et al.).

Collectively, the above findings indicate that health education level is one of the key factors in improving health outcomes. Then, how can health education be effectively promoted on a broader scale? It is well-accepted that due to the strong learning abilities, college students play a crucial role in the dissemination of health knowledge. In China, a separate study assessed the current knowledge, attitudes, and practices (KAP) among college students regarding foodborne diseases, an important public health issue. Contrary to expectations, these students had insufficient level of knowledge, moderate level of practices, but positive attitudes toward foodborne diseases (Ma et al.). Similar results have been observed in two additional large-scale KAP surveys on physical activity-related injuries (PARI) and bystander cardiopulmonary resuscitation (CPR) training. Results showed that students' attitudes toward PARI and CPR were, respectively, neutral and positive, which may be explained by varying levels of understanding (Kong, Zhu et al.; Qin et al.). However, most students still revealed low knowledge levels, highlighting the urgent need to implement public health educational interventions and allocate relevant resources in university environments. To address this challenge, Central South University innovatively applied the Delphi method to construct PARI-focused public health education programs for undergraduates. This initiative advances the development of health literacy and the establishment of proactive health management among young adults (Kong, Xu et al.).

Currently, the primary sources of health education encompass healthcare professionals, mass media, educational materials and school education (6). Among these, the media play an outstanding role in sharing healt-related information. In China, short-video platforms (e.g., TikTok, Bilibili, and Kwai) are experiencing rapid growth, owing to the sociality, interactivity, vividness and originality of short videos. Nevertheless, a cross-sectional study on the information quality of *H. pylori*-related videos across three platforms revealed that the content and quality of the videos were suboptimal (Lai et al.). Short videos produced by health professionals tend to offer more accurate, high-quality, and reliable guidance on disease prevention and treatment. In the future, short-video platforms should be refined to prevent the propagation of misinformation through online social networks. Furthermore, more professionals should be encouraged to release authoritative and credible short videos.

## 3 Public policy and health services

As the COVID-19 pandemic comes to an end, countries worldwide have shifted their public health policies from confronting the pandemic to enhancing the health of the entire population. Moreover, the onset of COVID-19 substantially accelerated the formulation and implementation of evidence-informed health policy regarding primary health care provision and community-based interventions.

With the support of relevant public policies, the National Institutes of Health (NIH) launched the Community Engagement Alliance (CEAL) during the early stages of the pandemic. In this program, communities serve as core components to facilitate collaboration between clinical researchers and social scientists (7). Through dynamic exchanges between community and academic experts, CEAL initiative has successfully fostered trust in science, countered misinformation, and advanced health equity in communities (Pons-Calvo et al.).

Consequently, the primary public health system has relatively improved in some countries. However, the majority of developing countries continue to encounter challenges in providing access to essential primary health services for economically disadvantaged and/or low-educated populations, especially in resource-limited settings. For example, Iraq's healthcare landscape has shifted from a hospital-centered model to a community clinic-centered service model. Nevertheless, significant inequalities in health-seeking behavior (HSB) impede individuals' access to high-quality primary healthcare (Mkhailef Hawi Al-Tameemi et al.). Similarly, evidence from Ethiopia shows that low maternal income and limited educational achievement hinder mothers' acceptance of healthcare professionals' recommendations, leading them to prefer receiving care from traditional healers at home rather than seeking more specialized medical services (Fenta Abebe et al.). As a result, when maternal referral is required, up to half of the referral practices prove unsatisfactory due to the lack of professional medical services, poor transportation access, and long travel time. Therefore, the government and public health officials can reduce health service disparities by designing more targeted and effective interventions to address economic and educational gaps, coupled with efforts to enhance individuals' health awareness.

## 4 Medical education reform

It has been widely acknowledged that the primary goal of medical education is to train qualified healthcare professionals who will improve the public health. However, medical education currently confronts a series of challenges such as insufficient training of emergency response ability, inadequate integration of interdisciplinary knowledge, and a lack of humanistic education (8). Accordingly, a growing number of medical education institutions have been carrying out reforms in higher medical education system.

### 4.1 Educational system reform

Prior to the pandemic outbreak in 2019, Chinese government proposed an important strategic plan “Healthy China 2030” aiming to improve national health literacy, reduce mortality, and extend healthy life expectancy. Within the framework of “Healthy China 2030” strategy, medical education is undergoing diverse reforms (Han and Wu).

Above all, the most crucial step is to shift orientation at new educational concepts, to secure the strong collaboration between public health and medical education. A growing body of research demonstrates the significance of incorporating public health into medical education. A meta-analysis of Ethiopian college students' understanding of COVID-19 underscores the need to implement targeted interventions to strengthen health education curricula and initiatives among students, thereby facilitating self-management of health and effective responses to pandemics (Chereka et al.). Consistent with these findings, a qualitative study on South African medical students indicates that incorporation of primary health care (PHC) re-engineering plan into undergraduate training helps to equip students with knowledge and skills in the field of PHC (Mabuza et al.).

In addition, the cultivation of humanistic qualities is recognized as an essential part of medical education, yet it has been neglected for a considerable period of time. To strengthen humanistic education for medical students, colleges have incorporated humanistic courses into the curriculum system. Furthermore, the implementation of labor education has been shown to help students establish a correct outlook in labor and develop a social consciousness of saving (Wang, Zhou et al.).

### 4.2 Teaching reforms

Following confirmation of the role of public health education in enhancing awareness and capacity for disease prevention, there is a need to seek effective strategies to integrate public health content into medical curricula. The University of Dundee Medical School has launched a medical curriculum reform by incorporating public health into the wider curriculum. The Bigger Picture provides context for professional development by replacing didactic sessions on healthcare with lectures, tutorials and workshops, and providing additional teaching materials in elective components within the course. Meanwhile, the Dundee Doctor serves as a framework for scrutinizing new proposals regarding teaching content and assessing learning outcomes (Hothersall).

As a cooperative learning strategy, the Jigsaw teaching method emphasizes group work and full engagement in learning activities. In contrast, the Self-Regulated Learning cycle encourages students' independent learning and self-monitoring. Notably, the combination of these two approaches can boost the interplay of personal, behavioral, and environmental factors, thereby more effectively improving students' self-regulated learning ability and commitment (Chen et al.).

Educational digitalization is a pivotal element of teaching reform. By utilizing modern information technologies such as human-computer interaction, cloud computing and big data analytics, the curriculum teaching can deliver intelligent learning guidance, a high-quality learning environment, and favorable learning conditions (9). For example, the application of virtual Reality (VR) technology in classroom settings can simulate real clinical scenarios, and offer personalized learning paths and resources. Consequently, this technology not only enhances students' learning motivation and practical skills, but also strengthens their self-confidence and professional competencies. Therefore, clinical nurse educators from a tertiary hospital in Wuhan city exhibit a strong willingness to adopt VR technology (Mengying et al.).

### 4.3 Reform of talent training and assessment

High-quality medical talents are the foundation for delivering satisfactory medical and health services. However, as public healthcare systems grow increasingly complex, the demands on medical talents have become more stringent in the new era. Beyond professional knowledge and skills, good humanistic literacy and self-learning ability, a qualified medical student should also possess innovative spirits and strong emotional regulation ability (10).

Against the backdrop of the pandemic, public health educators and practitioners have recognized that medicine is an evolving discipline. Only medical students equipped with innovative capabilities can actively explore optimal medical processes, introduce new technologies and methods, and improve treatment efficacy (11). Indeed, Chinese medical institutions have come to focus their attention on cultivation of high-quality public health talents with innovative capabilities. Moreover, Wang, Geng et al. constructed a nomogram to predict and evaluate the academic innovation level of public health students, thereby supporting more accurate and efficient training.

To meet patients' healthcare needs, medical students must devote greater effort to acquiring professional knowledge and skills, along with increased mental stress in their future clinical practice. Hence, mental health has emerged as an important health concern for medical students worldwide, especially in Asia where students are prone to mental health issues. An analysis of happiness perception among Korean medical students revealed that enhancing students' happiness is mainly dependent on social support, self-management, and quality of life (Lee et al.). In light of these findings, medical schools should foster students' wellbeing by providing opportunities for emotion regulation training, physical activities, and career guidance services.

Numerous universities have introduced the Health Promoting Universities (HPUs) initiative to create an environment that integrates health (physical and mental) into institutional culture, policies, and activities (12). In the context of HPUs, all members (students, teachers and staff) have a high level of participation in the decision-making processes regarding health that influence their learning, working and living. Nevertheless, the successful adaptation of HPUs to local culture is essential for the effective implementation of HPU initiatives (Boncheva et al.).

Last but not least, the reliable and valid assessment is of considerable importance to comprehensive education reform, helping to determine whether a reform is working and how to implement improvements. At present, assessment methods have shifted from summative to formative assessment (13). Formative assessment is used to monitor students' progress to provide ongoing feedback that supports the continuous development of their comprehensive qualities and abilities. A cross-sectional study shows that the perceived formative assessment can directly boost psychological empowerment, further foster positive academic emotions, and ultimately promote medical students' learning autonomy (Wang, Zhang et al.).

In summary, the explosive growth of health education has contributed to significant advancements in public health as manifested by improved public health awareness, enhanced public health outcomes, and strengthened public health system. However, we must clearly recognize that the growth in public health lags behind the growth in demand, which has been well-substantiated by the COVID-19 pandemic.
